# Teicoplanin pharmacokinetics in critically ill patients on extracorporeal organ support: a retrospective analysis

**DOI:** 10.1186/s40635-025-00729-9

**Published:** 2025-02-21

**Authors:** Giovanni Camen, Pedro David Wendel-Garcia, Rolf Erlebach, Mattia Müller, Caroline John, Alix Buhlmann, Rea Andermatt, Reto A. Schuepbach, Sascha David, Daniel A. Hofmaenner

**Affiliations:** Institute of Intensive Care Medicine, University Hospital Zurich, Raemistrasse 100, 8091 Zurich, Switzerland

**Keywords:** Teicoplanin, Therapeutic drug monitoring, Extracorporeal membrane oxygenation

## Abstract

**Background:**

Extracorporeal membrane oxygenation (ECMO) can alter the pharmacokinetics of diverse antimicrobials, posing challenges in achieving therapeutic drug levels. Some literature suggests that teicoplanin may require higher dosing in ECMO patients, however the respective evidence is scarce. The aim of this study was to assess teicoplanin trough levels in critically patients on ECMO support and to compare patients with and without additional continuous renal replacement therapy (CRRT). We conducted a retrospective study at the Intensive Care Unit (ICU) of the University Hospital Zurich, Switzerland. Teicoplanin trough levels and doses were analyzed in critically ill patients during ECMO support by means of a non-parametric local estimated polynomial regression. Outcomes included the proportion of patients with insufficient or toxic teicoplanin trough levels, dosage adjustments, and differences in teicoplanin trough levels between patients with and without additional CRRT during ECMO support.

**Results:**

After screening 172 patients receiving teicoplanin therapy during their ICU stay from 1.1.2020 to 19.07.2023, a total of 23 adult patients were included. The proportion of patients with insufficient teicoplanin levels was notably higher during ECMO support compared to patients with toxic levels (78.3% vs. 13% of patients, respectively). Teicoplanin dosages mostly were increased during the first few days of ECMO treatment. Concomitant CRRT led to a further increase in the proportion of patients with insufficient levels.

**Conclusions:**

Teicoplanin trough levels using standard dosing tend to be low in patients on ECMO support, especially in the early days of therapy. Higher doses than the standard regimen are often necessary to achieve therapeutic levels, particularly in patients receiving additional CRRT.

**Supplementary Information:**

The online version contains supplementary material available at 10.1186/s40635-025-00729-9.

## Background

Extracorporeal membrane oxygenation (ECMO) is an increasingly utilized life-support modality for critically ill patients with respiratory or circulatory failure [1, 2]. However, despite its life-saving potential and improved management strategies, ECMO therapy can expose clinicians to several management challenges and complexities. Over the past several years, attention has been drawn to the potential impact of ECMO circuits on drug pharmacokinetics [3, 4]. The ECMO circuit, particularly the oxygenator, can change the pharmacokinetics of some administered drugs including antimicrobials, e.g., through splicing [3]. Furthermore, volume of distribution and endogenous drug clearance may be altered. These changes thus may have significant implications for the efficacy of medications such as antibiotics and antifungals, potentially resulting in inadequate treatment or even treatment failure.

Previous studies have demonstrated that substances, such as, e.g., voriconazole and isavuconazole exhibit lower trough levels in patients receiving ECMO support, potentially compromising their therapeutic effect [5–8]. Consequently, regular therapeutic drug monitoring (TDM) might be a critical tool for guiding antimicrobial therapy in this patient population [8]. Nevertheless, clear guidelines on antimicrobial stewardship and antimicrobial TDM in patients undergoing extracorporeal organ support are limited.

Teicoplanin, a hydrophilic, protein-bound glycopeptide antibiotic widely utilized to treat Gram-positive infections, has been used in the critical care setting in particular in the immunocompromised patient [9, 10]. It is sometimes favored over vancomycin due to its presumed lower nephrotoxicity and reduced incidence of adverse effects. However, emerging evidence suggests that teicoplanin, like other antibiotics, may display lower serum levels in patients on ECMO support [11]. While previous studies indicate the need for higher dosing of teicoplanin during ECMO support, most of these investigations have been limited by very small sample sizes, leaving a gap in the understanding of optimal dosing strategies [3, 11, 12].

The aim of our study was to analyze teicoplanin levels and administered doses in a cohort of critically ill patients receiving ECMO support ± CRRT. We hypothesized that a relevant amount of patients would have sub-therapeutic trough levels on ECMO.

## Methods

### Study design and population

This single-center retrospective study included critically ill, adult (> 18 years old) patients hospitalized at the tertiary intensive care unit (ICU) of the University Hospital Zurich, Switzerland, an academic teaching hospital. The local ICU provides care for patients from all medical and surgical fields. All patients undergoing extracorporeal membrane oxygenation (ECMO) support and intravenous teicoplanin treatment were eligible for study inclusion from 1.1.2010 to 19.07.2023. Patients were included irrespective of the admitting diagnosis and independent of the ECMO configuration (veno-venous, veno-arterial or others with, e.g., multiple cannulation sites). Patients aged < 18 years were excluded. Other exclusion criteria were documented verbal or written refusal to participate in this study. Patients were also excluded if teicoplanin was not administered for any portion of the ECMO treatment period (e.g., if given only before or after the ECMO treatment period) or if no trough levels were obtained during ECMO support.

The study was conducted according to the principles of the Helsinki Declaration and was approved by the competent local Ethics committee (Cantonal Ethics Commission Zurich, BASEC Number 2023-01379).

### Data collection

Data were collected with the use of two in-hospital electronic medical records databases, the KISIM (Cistec AG, Zurich, Switzerland) and the Patient Data Management System (PDMS) MetaVision (iMDsoft, Dusseldorf, Germany). Collected data included baseline demographic data (including age, gender and body mass index), comorbidities (including cardiovascular conditions, pulmonary diseases, renal insufficiency) and immunosuppression (including patients with prior organ transplantation), ICU admitting diagnoses, and the indication for teicoplanin treatment (empiric vs. targeted). Moreover, ICU treatment modalities and scores (e.g., organ support and scores such as the Sequential Organ Failure Assessment Score SOFA and the Simplified Acute Physiology Score SAPS II) and patient outcomes (including ICU length of stay and survival) were gathered. Teicoplanin trough levels and dosages over time were obtained from the medical records (see below).

### Teicoplanin prescribing policy and assessment

In our hospital, intravenous teicoplanin is normally administered for empiric or targeted coverage of Gram-positive microorganisms. The standard loading dose is 400 mg infused at 0 h, 12 h and 24 h. Thereafter, 400 mg are administered every 24 h. Measurement of teicoplanin trough levels (T_Ctrough_) is recommended prior to the fifth dose and this also applies to dose adjustments. In case of renal impairment, T_Ctrough_ levels are recommended to be measured prior to the second dose, in order to detect potential overdosing early. If the estimated glomerular filtration rate (eGFR) is subnormal, teicoplanin starting dosage is adjusted (eGFR 40–60 ml/min: 200 mg/24 h, eGFR < 40 ml/min: 100 mg/24 h).

In case the patient is undergoing continuous renal replacement therapy, 200–400 mg teicoplanin every 24 h are recommended. In general, T_Ctrough_ of 10–30 mg/l are targeted. T_Ctrough_ < 10 mg/l are considered insufficient [13], whereas T_Ctrough_ > 30 mg/l are considered toxic. In case T_Ctrough_ are not within the target ranges, dose adaptations can be prescribed by the clinicians in charge. In case a measured T_Ctrough_ is toxic (i.e., > 30 mg/l), no more teicoplanin is administered, however T_Ctrough_ are measured on a daily basis. As soon as the teicoplanin levels return to the normal range (i.e., 10–30 mg/l), teicoplanin is reinstituted in a lower dose according to the clinicians in charge. In case T_Ctrough_ are subnormal (i.e., < 10 mg/l), the dose is increased according to the clinicians in charge taking pharmacokinetic considerations and renal function (or replacement therapy) into account. After increasing the dose, subsequent teicoplanin levels are again measured prior to the fifth dose or prior to the second dose in case of renal impairment, respectively.

Owing to lacking data, there is no specific in-house protocol for laboratory T_Ctrough_ assessments in patients undergoing ECMO support. In order to reflect real-world practice and to evaluate the potential impact of ECMO treatment on teicoplanin levels, T_Ctrough_ were assessed as available from the medical records, beginning up to one week before ECMO implantation (when available) and continuing throughout the ECMO treatment period.

### Continuous renal replacement therapy

As a standard, the multiFiltrate CRRT device with AV1000 membranes (mFT, Fresenius Medical Care, Bad Homburg, Germany) is used in our institution. The standard configuration is continuous veno-venous hemofiltration with regional citrate anticoagulation. In case of documented citrate accumulation, we switch to heparin anticoagulation or continuous veno-venous hemodiafiltration without citrate administration, depending on the presumed bleeding risk of the patient. An initial dialysis dosage of 20–25 ml/kg/h and a blood flow:dialysate ratio of 1:20 is targeted, which both can be adapted according to the clinical circumstances such as acid–base conditions. In case fluid removal is intended, fluid is directly removed via the dialysis device according to the clinicians in charge.

### Study outcomes

The primary outcome of this study was the proportion of patients with insufficient or toxic T_Ctrough_ during ECMO support. Secondary outcomes included the observed T_Ctrough_ and teicoplanin dosages over time during ECMO support and dependent on the presence of continuous renal replacement therapy (CRRT), as well as the occurrence of side effects (such as hemotoxicity, hepatotoxicity or skin changes) leading to discontinuation of teicoplanin treatment.

### Statistical analysis

Data were expressed as median and interquartile ranges IQR (25th−75th percentile) for continuous variables or as absolute numbers and percentages for categorical variables, as appropriate.

T_Ctrough_ and teicoplanin dosages over time were modeled by means of a non-parametric local estimated polynomial regression with a tri-cubic weighting function and an alpha of 0.75, including the presence of CRRT. Statistical analysis was performed through a fully scripted data management pathway using the R environment for statistical computing version 4.2.1. A two-sided *p* < 0.05 was considered statistically significant.

## Results

In total, 172 patients with teicoplanin treatment during the ICU stay were screened for inclusion. Of these, 27 received teicoplanin during ECMO treatment. After excluding minor patients (< 18 years old), 23 patients were included in the final analysis.

In Table 1, baseline characteristics and ICU treatment modalities of included patients (*n* = 23) are demonstrated. 60.1% of patients were male, the median age was 49 years (interquartile range IQR 41–57 years). Median Sequential Organ Failure Assessment (SOFA) scores at ICU admission and at ECMO cannulation were 5 (IQR 4–8) and 6.5 (IQR 4–10.5), respectively (Table 2). Most of patients had a veno-venous ECMO configuration (65.2%), with a median ECMO runtime of 12 days (IQR 6–31 days).

**Table 1:** Baseline characteristics at ICU admission and laboratory values at ECMO initiation

Baseline characteristics	
Male gender	14 (60.1%)
Age (year)	49 (41–57)
BMI (kg/m^2^)	21 (18.4–25.2)
Obesity (BMI > 30 kg/m^2^)	1 (4.3%)
Arterial hypertension	7 (30.4%)
Chronic heart failure	5 (21.7%)
Chronic kidney disease	5 (21.7%)
Cerebrovascular disease	0 (0%)
Obstructive lung disease	6 (26.1%)
Liver disease (cirrhosis)	0 (0%)
Diabetes type I/II	8 (34.8%)
Solid organ transplantation	7 (30.4%)
Immunosuppression	12 (52.2%)
Active cancer	1 (4.3%)
Alcohol abuse	2 (8.6%)
Active smoking	4 (17.4%)

Laboratory values at ECMO initiation	
C-reactive protein (mg/l)	91 (34–236.5)
Procalcitonin (μg/l)	0.62 (0.16–3.24)
Sodium (mmol/l)	140 (137–142)
Creatinine (μmol/l)	82 (56.3–123.5)
Urea (mmol/l)	9.5 (5.9–15.3)
eGFR (ml/min)	73 (44.5–90)
Albumin (g/l)	26.5 (24–31)
Bilirubin (μmol/l)	9.5 (4.3–20.5)
AST (U/l)	37.5 (24.3–70)
ALT (U/l)	32 (17.3–73)
ALP (U/l)	105.5 (79.3–157)
LDH (U/l)	479.5 (362.3–707.8)
Hemoglobin (g/l)	87.5 (75.8–105.3)
Leukocytes (G/l)	11.85 (8.8–16.8)
Platelets (G/l)	255 (115.3–377.5)
INR	1.1 (1–1.3)
Lactate (mmol/l)	0.9 (0.7–1.6)

Categorical data are presented in numbers (percentages) and numerical data in median (interquartile range), as appropriate

ECMO: extracorporeal membrane oxygenation; BMI: body mass index; eGFR: estimated glomerular filtration rate; AST: aspartate aminotransferase; ALT: alanine aminotransferase; LDH: lactate dehydrogenase; INR: international normalized ratio

**Table 2:** ICU characteristics and outcomes

ICU characteristics	
Admission diagnosis	
* Sepsis*	1 (4.3%)
* ARDS*	1 (4.3%)
* Cardiogenic shock*	3 (13%)
* Acute respiratory failure*	10 (43.5%)
* Admission for planned organ transplant*	5 (21.7%)
* Others*	3 (13%)
SAPS II score	36 (24–61)
SOFA score at ICU admission	5 (4–8)
SOFA score at ECMO initiation	6.5 (4–10.5)
ECMO modality	
* Veno-venous*	15 (65.2%)
* Veno-arterial*	7 (30.4%)
* Others*	1 (4.3%)
ECMO runtime (d)	12 (6–31)
Number of oxygenator changes (*n*)	0 (0–2)
Mechanical ventilation	21 (91.3%)
CRRT	10 (43.5%)
Vasoactives	22 (95.7%)
Inotropes	6 (26.1%)
ICU mortality	7 (30.4%)
ICU length of stay (d)	39 (15–60)

Categorical data are presented in numbers (percentages) and numerical data in median (interquartile range), as appropriate

ICU: intensive care unit; ARDS: acute respiratory distress syndrome; SAPS II: Simplified Acute Physiology Score II; SOFA score: Sequential Organ Failure Assessment Score; CRRT: continuous renal replacement therapy

Almost all patients were mechanically ventilated (91.3%) and treated with vasoactive drugs (95.7%) (Table 2). Ten out of 23 patients (43.5%) underwent continuous renal replacement therapy (CRRT). The median eGFR of patients not receiving CRRT during ECMO support (*n* = 13 patients) was 49 ml/min (IQR 21.5–85.5 ml/min). According to the Kidney Disease Improving Global Outcomes Acute Kidney Injury (KDIGO AKI) classification, two patients did not have AKI (2/13, 15.4%), four patients had AKI stage I (4/13, 30.8%), three patients AKI stage II (3/13, 23.1%), and four patients AKI stage III (4/13, 30.8%), respectively. The median ICU length of stay was 39 days (IQR 15–60 days), and around 30% of the patients (7/23) died in the ICU (Table 2).

Teicoplanin was mostly used empirically (82.7%) and not targeted, the median length of teicoplanin application was 12 days (IQR 7–23 days) (Table 3). In most of the patients (13/23, 56.5%), teicoplanin was started during ECMO therapy and not prior to ECMO cannulation (Table 3). Six patients (26.1%) had already been treated with teicoplanin prior to ICU admission. While almost 80% of the patients (78.3%) had at least one insufficient T_Ctrough_ (T_Ctrough_ < 10 mg/l) during ECMO treatment, T_Ctrough_ was in a potentially toxic range (T_Ctrough_ > 30 mg/l) in only 3 patients (13%).

**Table 3:** Characteristics of teicoplanin administration

Teicoplanin indication	
* Targeted*	4 (17.4%)
* Empiric*	19 (82.7%)
Teicoplanin use before ICU admission	5 (21.7%)
Teicoplanin start on ICU before ECMO	5 (21.7%)
Teicoplanin start during ECMO	13 (56.5%)
Toxic dose during ECMO (> 30 mg/l)	3 (13%)
Insufficient dose during ECMO (< 10 mg/l)	18 (78.3%)
Teicoplanin dose increased during ECMO	11 (47.8%)
Teicoplanin dose decreased during ECMO	2 (8.6%)
Teicoplanin treatment (d)	12 (7–23)

Categorical data are presented in numbers (percentages) and numerical data in median (interquartile range), as appropriate. The identified microorganisms corresponding to the targeted teicoplanin indication were *Staphylococcus epidermidis* (*n* = 3) and *Enterococcus faecium* (*n* = 1)

ECMO: extracorporeal membrane oxygenation.

**Fig. 1:**
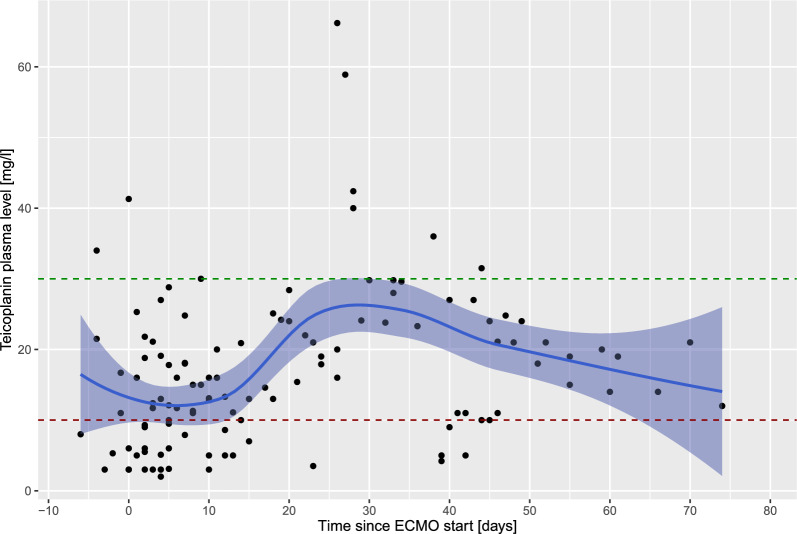
Teicoplanin plasma trough levels (T_Ctrough_) over time. Day 0 is defined as the day of ECMO cannulation. Available T_Ctrough_ were analyzed from one week prior to ECMO cannulation. Black dots represent individual T_Ctrough_. T_Ctrough_ < 10 mg/l are considered insufficient, whereas T_Ctrough_ > 30 mg/l are considered toxic. The green and red dotted lines represent the normal range for teicoplanin levels

**Fig. 2:**
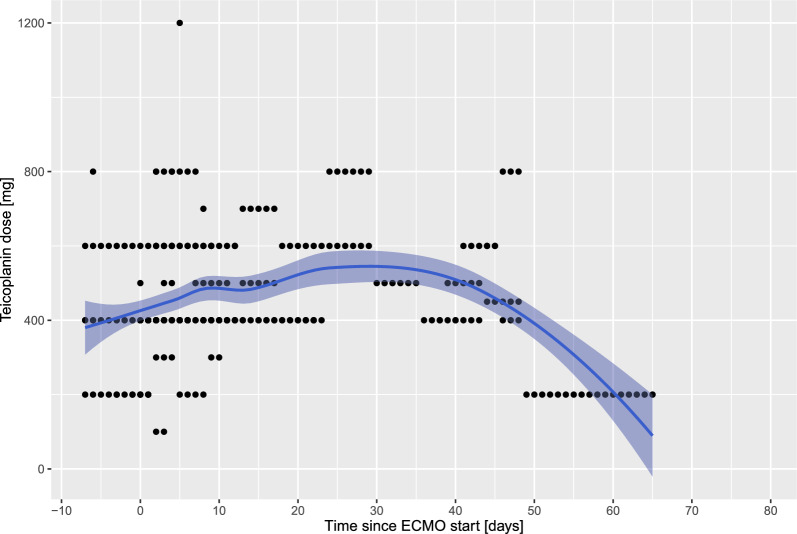
Teicoplanin doses over time. Day 0 is defined as the day of ECMO cannulation. Available teicoplanin doses were analyzed from one week prior to ECMO cannulation

**Fig. 3:**
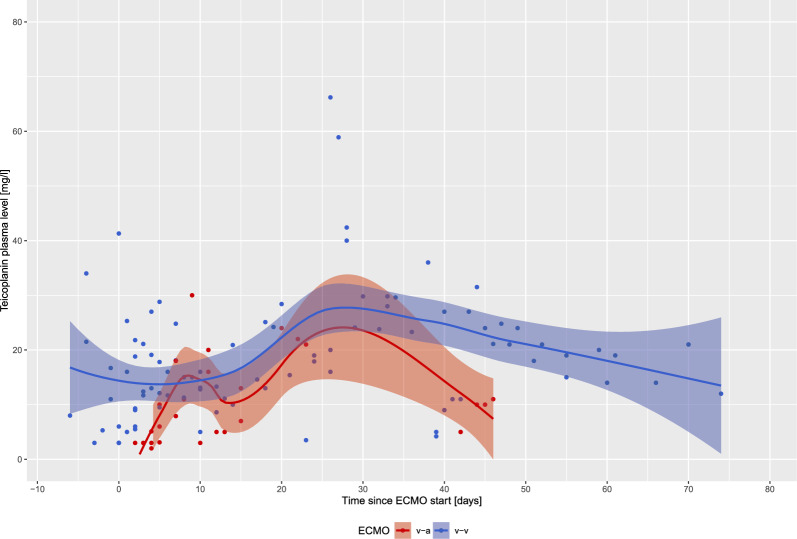
Teicoplanin plasma trough levels (T_Ctrough_) over time depending on the configuration of the ECMO circuit (veno-venous vs. veno-arterial). Day 0 is defined as the day of ECMO cannulation

Figure 1 shows T_Ctrough_ over time during ECMO treatment with numerous insufficient levels (T_Ctrough_ < 10 mg/l) particularly during the first 14 days on extracorporeal support. Thereafter, T_Ctrough_ become more homogenously distributed (steady-state like). Correspondingly, overall teicoplanin doses were increased during the first days of ECMO treatment, with gradual dose reductions after 2–3 weeks of extracorporeal support (Fig. 2). Individual single lines of all patients demonstrating T_Ctrough_ and teicoplanin doses are reported as Supplemental Fig. 1. Correlation between teicoplanin dose and T_Ctrough_ was not significant (*R* = 0.066, *p* = 0.53) (Supplemental Fig. 2). The configuration of the ECMO circuit (veno-venous vs. veno-arterial) did not have a significant influence on T_Ctrough_ over time (Fig. 3).

In addition, the impact of ECMO oxygenator changes on T_Ctrough_ was calculated. We found a T_Ctrough_ mean difference of 1.5 mg/l ([95% CI – 12 to 15], *p* = 0.8108) in relation to oxygenator changes (pre vs. post) from five cases where respective data were available (Supplemental Fig. 3).

**Fig. 4:**
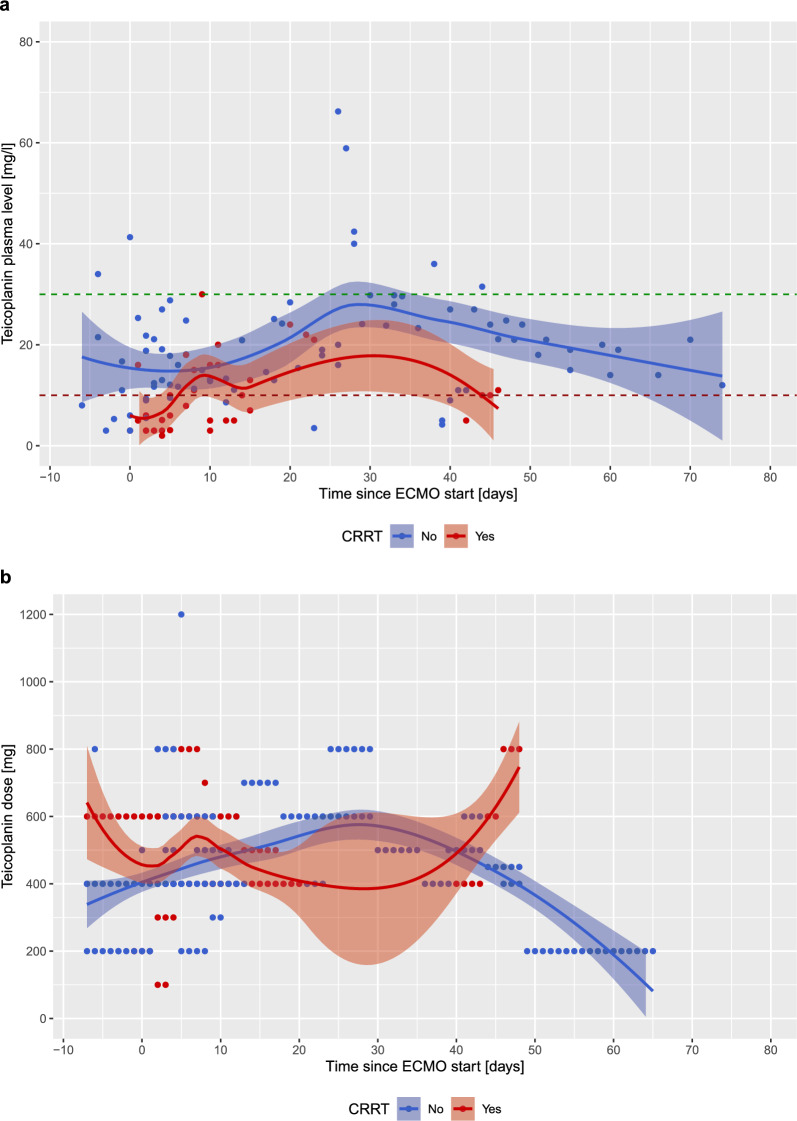
T_Ctrough_ (**a**) and teicoplanin doses (**b**) according to continuous renal replacement therapy. T_Ctrough_ < 10 mg/l are considered insufficient, whereas T_Ctrough_ > 30 mg/l are considered toxic. The green and red dotted lines represent the normal range for teicoplanin levels

Figure 4 shows T_Ctrough_ and teicoplanin doses stratified for CRRT. Patients with CRRT exhibited lower T_Ctrough_ over the whole ECMO period, and needed higher teicoplanin doses during the first days of ECMO support (Fig. 4). In patients with ECMO treatment alone, T_Ctrough_ were 18 mg/l [IQR 12–24 mg/l], whereas in patients with ECMO and CRRT, T_Ctrough_ were 9 mg/l [IQR 5–16], corresponding to a mean difference of 9 mg/l [95% CI 6–13], p < 0.0001.

In all included patients, no drug reactions or other adverse reactions to teicoplanin were observed.

## Discussion

In this study, we assessed teicoplanin levels and dosages in critically ill patients receiving ECMO therapy.

We found that the proportion of patients with insufficient teicoplanin levels exceeded the number of patients with toxic dosages during extracorporeal support. Furthermore, T_Ctrough_ were frequently insufficient during the first days of ECMO support, and doses were increased by clinicians subsequently.

Recently, more attention has been drawn to antimicrobial serum levels and dosing in patients receiving ECMO treatment [3]. While marked concentration variability during ECMO treatment has been observed for many common antimicrobials used in the ICU including meropenem, cefepime, ceftriaxone, piperacillin or vancomycin [14], data for teicoplanin are scarce so far. In a population pharmacokinetic model for dose optimization including 10 adult ECMO patients, Wi et al. found that despite a 34% lower central volume of distribution, ECMO treatment was associated with reduced probability of target attainment [11]. For severe infections, the authors suggested a loading dose of 1000 mg and maintenance dose of 800 mg for patients without renal replacement therapy (CRRT) [11]. In patients undergoing RRT, the recommended doses were even higher [11]. Those findings are in line with this study, where especially during the first days of ECMO therapy T_Ctrough_ were frequently insufficient, and doses were subsequently adjusted by the ICU doctors. As the phenomenon of sequestration within the ECMO circuit has so far been mainly characterized for lipophilic drugs such as voriconazole and isavuconazole [7, 15], other factors such as altered protein binding, hemodilution or further pathophysiologic changes occurring in ECMO patients might be more relevant for the lower trough levels of the hydrophilic drug teicoplanin. However, in a recent ECMO ex vivo study, there was a significant loss of teicoplanin within the ECMO, suggesting that also the ECMO circuitry likely contributes to our observed results [16]. Potential reasons might include absorption by the cannulas or sequestration within the ECMO oxygenator. Interestingly, the observed effects differed according to different blood-primed ECMO circuits, making external effects of particular ECMO material very likely [16].

Chen et al. focused on the effect of applying high loading doses in 11 patients receiving veno-arterial ECMO treatment [12]. In their study, four loading doses greater than 10 mg/kg were applied within the first 72 h [12]. Before the application of the maintenance dose, the authors reported adequate therapeutic T_Ctrough_ for severe infections in more than 90% of patients [12]. In our study, the loading dose of 400 mg was markedly lower, which presumably might have contributed to our observed low T_Ctrough_ in a significant proportion of patients particularly during the first days of ECMO support.

As teicoplanin is highly hydrophilic, it is mainly cleared by the kidneys. Evidence from previous literature suggests that during CRRT teicoplanin needs to be dosed even higher in ECMO patients [11]. These findings align with our results, where T_Ctrough_ were lower over the whole ECMO treatment period in patients on CRRT. Moreover, as calculated per mean difference, T_Ctrough_ were substantially lower in patients with ECMO and CRRT compared to patients with ECMO treatment alone. Special attention should thus be given in ECMO patients with concomitant renal failure undergoing CRRT, as teicoplanin might need to be administered in higher doses in order to achieve therapeutic levels. On the other hand, in our cohort, only one of the patients with a toxic teicoplanin dose during ECMO support had chronic kidney disease, but did not undergo CRRT.

Our findings are in accordance with recent literature on teicoplanin management in critically ill patients in general, where higher loading and maintenance doses appear to be necessary in a substantial part of the ICU population, irrespective of ECMO treatment [17–19]. ICU clinicians thus should be aware of the potential of underdosing teicoplanin also in critically ill patients without extracorporeal support. A possible solution would be dosing of teicoplanin according to body weight (mg/kg) with regular TDM assessments.

Future studies should include higher patient numbers and assess T_Ctrough_ prospectively and at predefined time points during ECMO support. To assess potential clearance by the oxygenator, T_Ctrough_ should be measured pre- and post-oxygenator within the ECMO circuit. In our analysis, T_Ctrough_ after an oxygenator change appeared to be lower in a limited number of cases with available data, although statistically insignificant. This raises the clinically important question whether single additional teicoplanin doses should be administered after ECMO oxygenator changes. This issue should be addressed in future prospective trials.

Insights from such research and regular TDM might help critical care clinicians in the future to individualize and tailor teicoplanin dosing in critically ill patients on extracorporeal support.

Our study has to account for some limitations. First, the study included a limited patient number. However, the combination of teicoplanin administration plus simultaneous ECMO treatment is relatively rare. Thus, the study yields clinically relevant findings for ECMO centers nevertheless. Second, the study was conducted in a single-centric design. The findings might thus not be transferable to other ECMO centers. Third, teicoplanin doses were not normalized to patient weight, potentially introducing biases.

Finally, owing to the retrospective nature, T_Ctrough_ were not assessed in a protocolled manner, which should be done in further prospective studies.

## Conclusions

In conclusion, teicoplanin levels are frequently low during the first days on ECMO therapy, with a more pronounced effect in patients on CRRT. The proportion of patients with insufficient teicoplanin levels exceeds the number of patients with toxic dosages during extracorporeal support, mandating TDM in this patient cohort.

## Supplementary Information

Supplementary Material 1: Supplementary Figure 1. Individual single lines of all patients demonstrating T_Ctrough_ and Teicoplanin doses over time during ECMO support. Day 0 is defined as the day of ECMO cannulation.

Supplementary Material 2: Supplementary Figure 2. Correlation between Teicoplanin dose and T_Ctrough_.

Supplementary Material 3: Supplementary Figure 3. T_Ctrough_ mean difference in relation to oxygenator changes.

## Data Availability

The datasets used and/or analyzed during the current study are available from the corresponding author on reasonable request.

## Abbreviations

CRRT: Continuous renal replacement therapy
ECMO: Extracorporeal membrane oxygenation
eGFR: Estimated glomerular filtration rate
ICU: Intensive care unit
IQR: Interquartile range
PDMS: Patient data management system
SAPS II Score: Simplified Acute Physiology Score II
SOFA Score: Sequential Organ Failure Assessment Score
T_Ctrough_: Teicoplanin trough level
TDM: Therapeutic drug monitoring
