# Supplementary figures and images for: Teicoplanin pharmacokinetics in critically ill patients on extracorporeal organ support: a retrospective analysis
Source: Intensive Care Med Exp. 2025 Feb 21;13:22. doi: 10.1186/s40635-025-00729-9 (PMC11845331; doi:10.1186/s40635-025-00729-9)

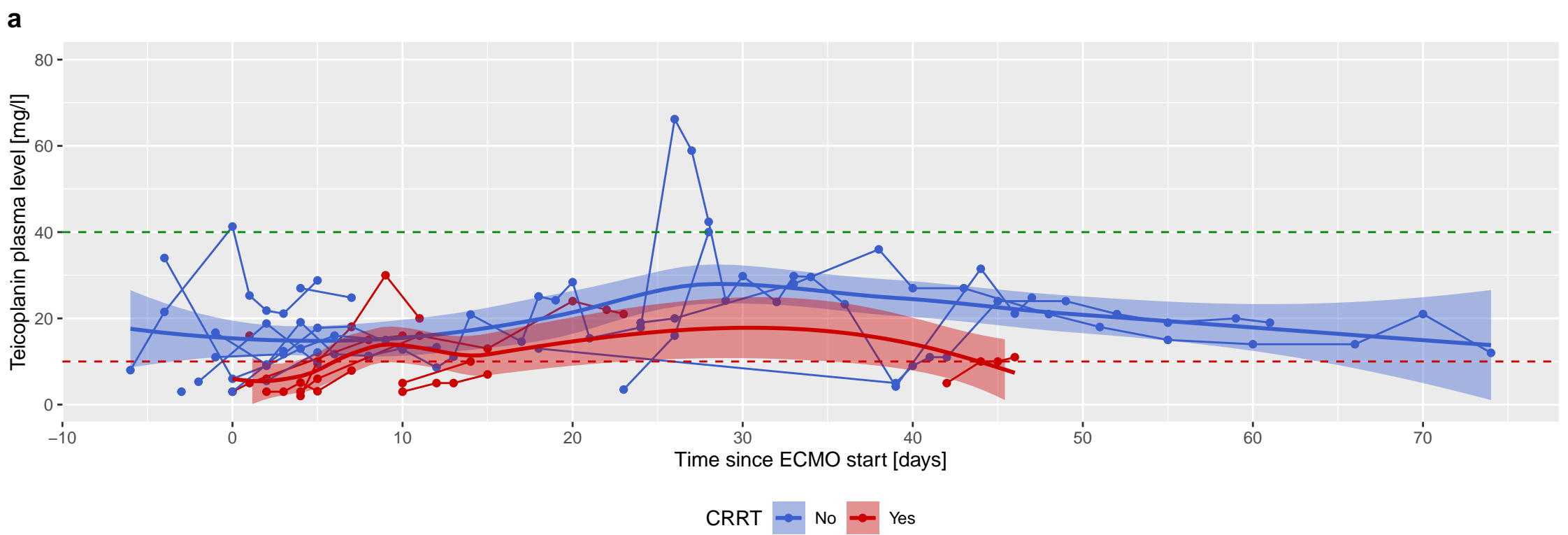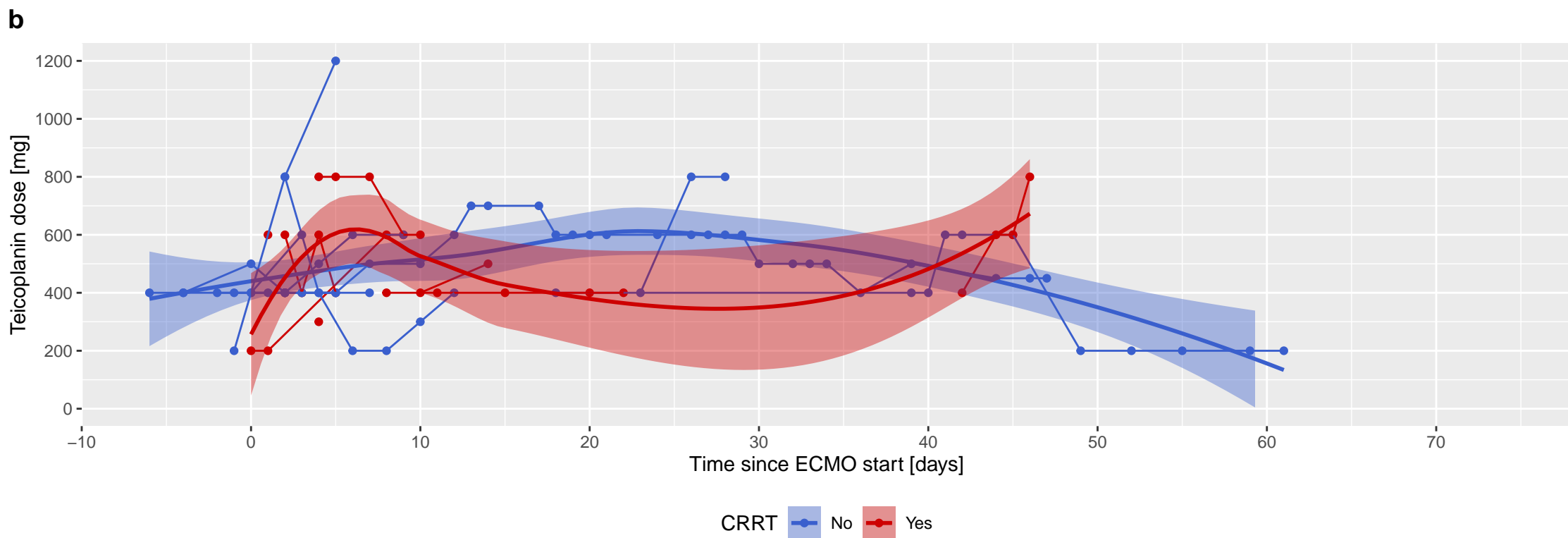

Supplement: Supplementary file 1 — Supplementary Material 1: Supplementary Figure 1. Individual single lines of all patients demonstrating TCtrough and Teicoplanin doses over time during ECMO support. Day 0 is defined as the day of ECMO cannulation. [file 40635_2025_729_MOESM1_ESM.pdf]

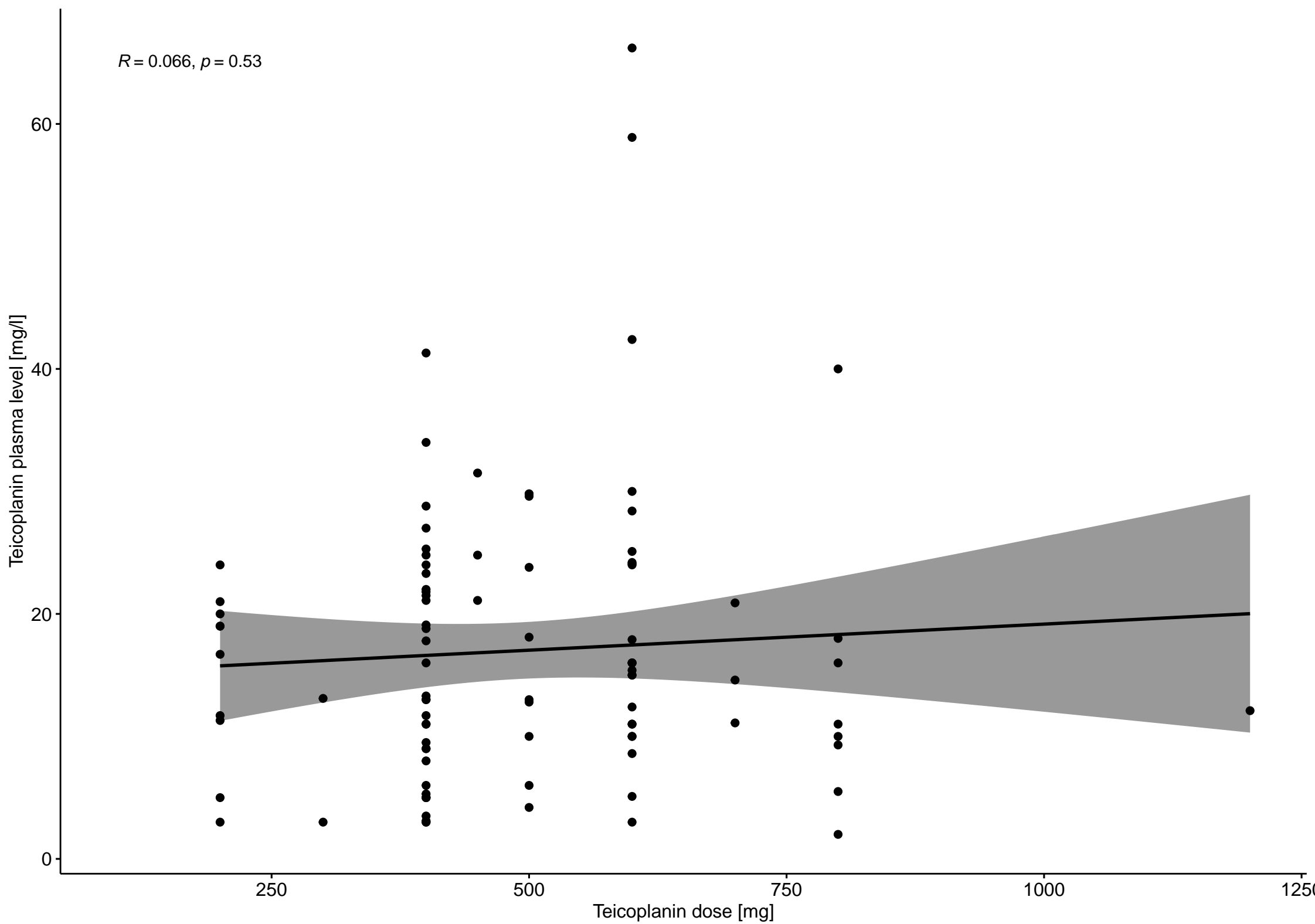

Supplement: Supplementary file 2 — Supplementary Material 2: Supplementary Figure 2. Correlation between Teicoplanin dose and TCtrough. [file 40635_2025_729_MOESM2_ESM.pdf]

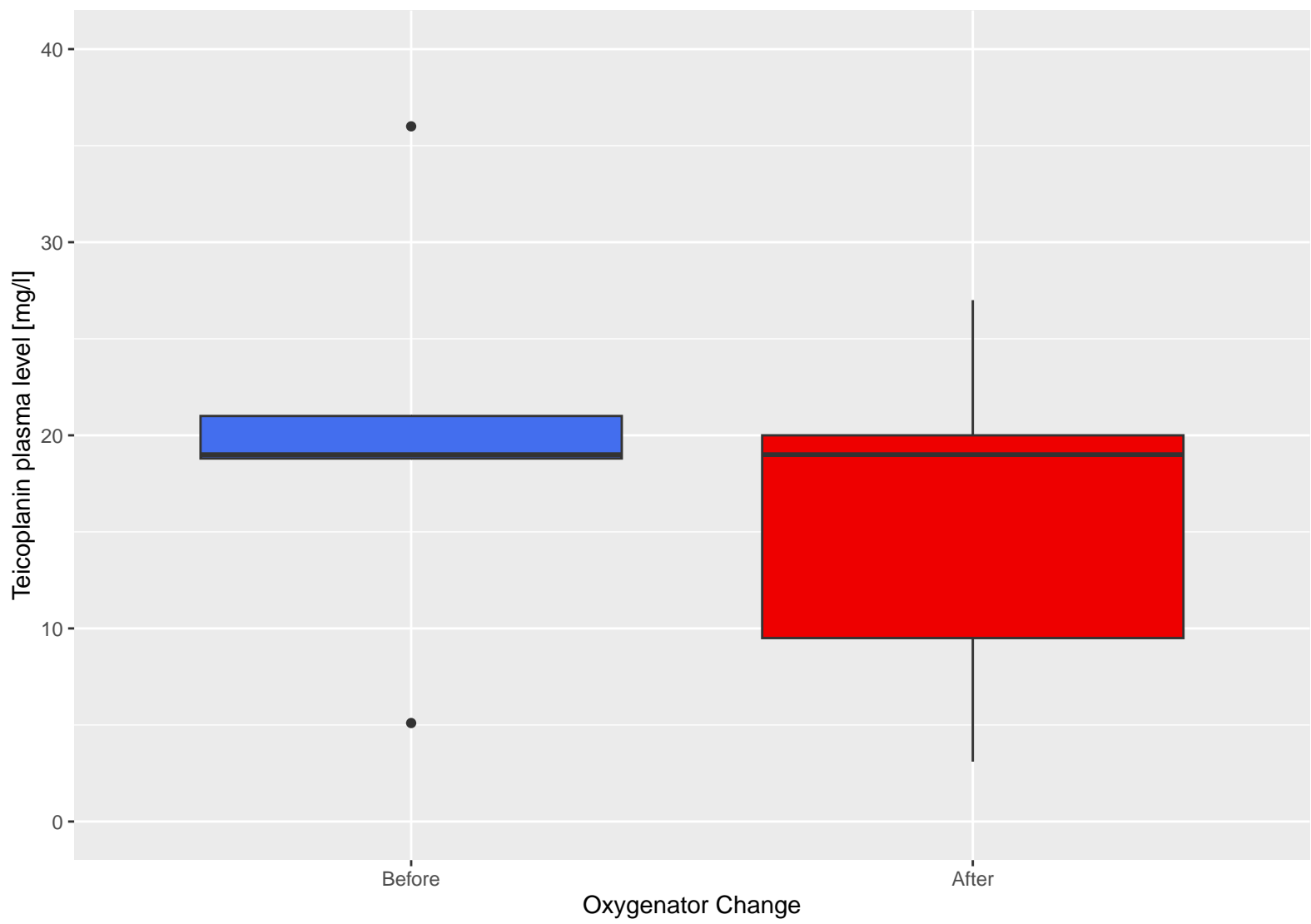

Supplement: Supplementary file 3 — Supplementary Material 3: Supplementary Figure 3. TCtrough mean difference in relation to oxygenator changes. [file 40635_2025_729_MOESM3_ESM.pdf]
